# A Convenient and Economical Spectrophotometric Assay for Ornithine Decarboxylase and Related Amino Acid Decarboxylases Using Sodium 2,4-Dinitrobenzenesulfonate

**DOI:** 10.1021/acsomega.5c12564

**Published:** 2026-06-10

**Authors:** Caihong Li, Sara Blankenship, Nikki Zheng, Robert S. Phillips

**Affiliations:** † Department of Chemistry, 1355 University of Georgia, Athens, Georgia 30602, United States; ‡ Department of Biochemistry and Molecular Biology, 138572 University of Georgia, Athens, Georgia 30602, United States

## Abstract

Ornithine decarboxylase (ODC) catalyzes the conversion of l-ornithine into putrescine as the first and rate-limiting step in polyamine biosynthesis. Common assays used to measure ODC activity are often costly, involve expensive reagents and instruments, and require labor-intensive protocols. To address these issues, this research developed an economical and user-friendly spectrophotometric assay for ODC and other decarboxylases. The principle of the assay relies on the reaction of the ODC product, putrescine, with sodium 2,4-dinitrobenzenesulfonate (DNBS) under alkaline conditions, with heating at 70 °C for 1 h, generating a bis-2,4-dinitrophenyl derivative that is extracted in toluene and measured spectrophotometrically at 340 nm, providing a sensitive readout of enzyme activity. This assay demonstrates decent sensitivity, with a limit of detection (LOD) for inducible ODC activity as low as 0.21 μg/mL (5.1 pmol), and kinetic analysis revealed a *K*_*m*_ of 0.34 ± 0.01 mM and *k*_*cat*_ = 32 ± 0.33 s^–1^. For the detection of putrescine, the product of ODC, the LOD was 4.8 nmol. Histamine detection by this method achieved an LOD of 18 nmol. This DNBS method was successfully applied to other decarboxylases, including bacterial histidine decarboxylase, d-ornithine/d-lysine decarboxylase (DOKDC) reaction with d-ornithine and a very slow substrate, d-arginine, and the very low activity C387A mutant DOKDC reaction with d-ornithine.

## Graphical abstract

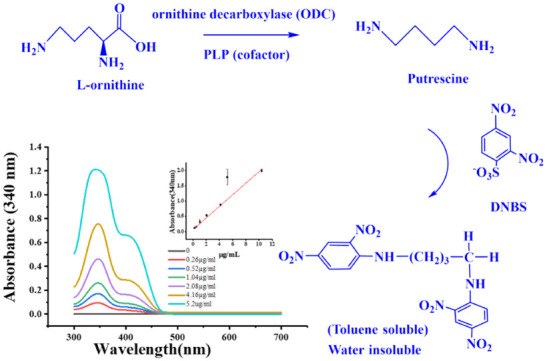

## Introduction

Decarboxylation of the basic aliphatic amino acids represents a fundamental biochemical pathway for the biosynthesis of polyamines,^1^ which is important for cell growth, DNA stabilization, protein synthesis,^2^ permeability of membranes, and biofilm formation.^3^ Furthermore, decarboxylase-mediated reactions play a key role in the bacterial acid stress response, in which an enzyme catalyzes a proton-dependent decarboxylation, releasing CO_2_ and consuming a proton, thereby contributing to the regulation of intracellular pH.^4^ In *Escherichia coli* and related enterobacteria, such as *Salmonella enterica*, basic amino acid decarboxylation is mediated by pyridoxal-5-phosphate (PLP)-dependent enzymes, existing as inducible and constitutive pairs for lysine (LdcI/LdcC), arginine (AdiA/SpeA), and ornithine (SpeF/SpeC).^5^ Ornithine decarboxylase (ODC, EC 4.1.1.17) converts l-ornithine to putrescine, which is the rate-limiting step for the synthesis of polyamines, spermine, and spermidine. The inducible ODC from *Lactobacillus* counteracts the pH decrease associated with lactic acid production, suggesting a role for ODC in pH regulation. The crystal structure of ODC from *Lactobacillus* shows that it is a dodecamer, with the monomer exhibiting a PLP-dependent fold Type I.^6^ In contrast, eukaryotic ODC is a member of PLP-dependent fold Type III. The truncated mouse ODC is a homodimer, which is in fast equilibrium between monomer and dimer.^7^ Furthermore, overexpression of ODC and the resulting elevated polyamine levels have been linked to the development of numerous diseases, including cancer and Alzheimer’s disease.^8^ Meanwhile, ODC is regarded as an oncogenic enzyme and a potential therapeutic target for various pathologies.^9^

A radioactive assay using ^14^C-labeled l-ornithine was used to measure decarboxylase activity by trapping released ^14^CO_2_ on alkali-soaked filters, which are subsequently analyzed by scintillation counting.^10^ This assay for ODC detection has very high sensitivity; however, it is limited by the expense of radiolabeling, which is not environmentally friendly, and the tedious preparation time. A cheaper method for decarboxylase assays was developed by using coupled phosphoenolpyruvate carboxylase (PEPC)–malate dehydrogenase (MDH) to detect CO_2_ produced by decarboxylases like ODC. In this assay, CO_2_ is converted by PEPC into oxaloacetate, which is then reduced by MDH to malate using NADH, measuring the decrease in absorbance at 340 nm.^11^ This assay allows continuous monitoring of decarboxylase activity using a UV–visible spectrophotometer. Moreover, the assay can be performed using a commercially available kit for decarboxylase activity measurement.^12^ However, the method suffers from low sensitivity and requires rigorous removal of CO_2_ from all solutions to minimize high background signals. In addition, the coupling enzymes and reagents are costly, resulting in an estimated expense of approximately $1 per sample (with the commercial kit priced above $300). The total assay time is approximately 40–50 min.

In 1982, a sensitive and convenient method was proposed to detect lysine decarboxylase from *Escherichia coli*, which is based on the reaction of the product, cadaverine, with 2,4,6-trinitrobenzenesulfonic acid (TNBS). The product, 1,7-bis­(2,4,6-trinitrophenyl)­cadaverine, is extracted into toluene, and the absorbance is measured at 340 nm to quantify lysine decarboxylase activity.^13^ This TNBS assay has also been used to measure the activity of ODC,^14^ histidine decarboxylase (HDC),^15^ and d-ornithine/d-lysine decarboxylase.^16^ The most convenient form of TNBS is a solid sodium salt. However, because TNBS is potentially explosive in the solid state, sodium TNBS is no longer available on the market. Hence, only diluted solutions of TNBS (e.g., 5% in water) are available, but they are expensive ($100/g), resulting in a high cost per sample, even though the reaction is convenient, since samples take only about 30 min of preparation time.^13^ A phenol red pH indicator assay can also be used to measure ODC and other decarboxylases, following the absorbance increase at 560 nm associated with the color change from yellow to red at a pH around the pK_a_ of 7.4.^17^ These assays are rapid and continuous. However, this method must be performed in a weakly buffered solution and is insensitive, subject to interference, and difficult to quantify. Other decarboxylases have been assayed with other pH indicators, with pK_a_ values near their pH optima, since inducible ornithine decarboxylase from *Lactobacillus* *30a* showed a pH optimum at 5.8,^14^ and lysine and arginine decarboxylases from *E. coli* have pH optima at 5.7 ^15^ and 5.2,^16^ respectively.

To address the above issues, other researchers have proposed an indirect assay to measure ODC activity by putrescine oxidation, catalyzed by soybean amine oxidase (SAO), which produces H_2_O_2_. The H_2_O_2_ then reacts with 4-aminoantipyrine and phenol in the presence of horseradish peroxidase to form a highly colored complex, detectable at 505 nm.^18^ A fluorescent dye (DSMI) and a macrocyclic receptor (CB6) were later used to achieve a fluorescence assay for the detection of ODC. The principle was based on the weak binding of l-ornithine to CB6, keeping the bound DSMI fluorescent. When ODC converts l-ornithine to putrescine, the higher-affinity putrescine displaces DSMI, causing decreased fluorescence. This fluorescence decrease can be conveniently measured in microplates to monitor ODC activity and kinetics, with the use of known inhibitors (DFMO, EGCG) as controls,^18^ but this assay requires multiple reagents and enzymes, increasing cost and experimental complexity. High-performance liquid chromatography (HPLC) can also be used to measure putrescine. To detect the decarboxylase activity, the enzymatic reaction is quenched with HClO_4_, proteins are removed by centrifugation, and the amine-containing supernatant is derivatized prior to analysis. Several derivatization strategies are used, including benzoylation, dansylation, *o*-phthalaldehyde (OPA), and carbamoylation, followed by fluorescence or scintillation detection. These approaches allow accurate measurement of ODC activity and have been applied in other inhibitor screening and tissue-based assays.^19^ Furthermore, HPLC/Q-TOF MS of benzoylated polyamines enables the detection of very low polyamine levels in biological samples. Alternatively, mass spectrometry combined with dansylation and flow injection analysis (FIA) provides a fast and sensitive method for detection, with detection limits in the femtomole range. This approach has been successfully applied to measure putrescine production from ODC activity in cell extracts, with sensitivity as low as 0.05 nmol/mg protein.^20^ However, both assays require very expensive instrumentation and trained personnel, and involve complex data analysis.

The above-described analytical assays for detecting ODC or other decarboxylase activity often show significant drawbacks, including the high cost per sample, using coupling reactions, long sample preparation times, or expensive instruments. To address these limitations, this research paper developed an alternative *in vitro* assay for monitoring the activity of inducible ODC from*S. enterica*, and other amino acid decarboxylases. This method is based on the reaction of the amine product with sodium 2,4-dinitrobenzenesulfonate (DNBS) under alkaline conditions, with heating at 70 °C, producing a 2,4-dinitrophenylamine product soluble in toluene, which can be quantified by UV-visible spectrophotometry at 340 nm (Figure 1). In contrast to TNBS, DNBS is stable in solid form and much less expensive, ∼$3/g. Thus, the DNBS assay is simple, economical, and convenient, providing a practical approach for quantifying ODC and other amino acid decarboxylase activities.

**Figure 1:**
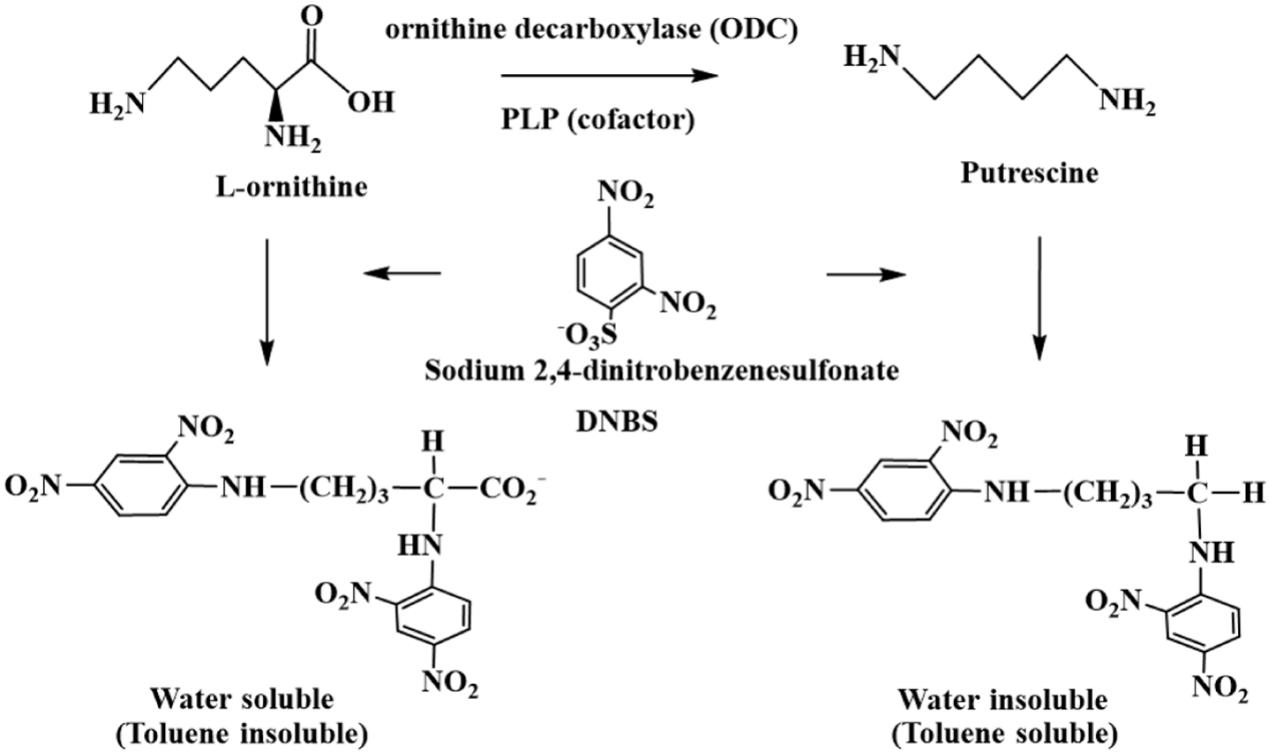
Schematic representation of the DNBS assay for decarboxylase activity detection.

## Materials and Methods

### Materials

Bis–tris, histamine hydrochloride, and putrescine dihydrochloride were obtained from Sigma-Aldrich. l-Ornithine was a product of Eastman, and 2,4-dinitrobenzenesulfonate (DNBS) sodium salt was purchased from TCI America, product code D0821. PLP was purchased from Ambeed. d-Ornithine, d-lysine, and d-arginine were obtained from AlfaAesar. Other chemicals were obtained from standard commercial sources.

### L-Ornithine Decarboxylase

The gene of inducible ODC from *S. enterica* serovar Typhimurium LT2 (SelF) was synthesized by Twist Bioscience, with codon optimization for*E. coli*, in pET-28b­(+). The gene was incorporated into the plasmid with an N-terminal hexaHis tag. Competent *E. coli* BL21­(DE3) cells (New England Biolabs) were transformed with the plasmid and plated on LB agar with 35 μg/mL kanamycin, followed by overnight incubation at 37 °C. A single colony was selected and used to inoculate a 5 mL overnight culture in LB supplemented with 100 μg/mL kanamycin. This culture was then transferred into 1 L of Studier autoinduction medium containing 400 μg/mL kanamycin.^21^ Cells were grown at 37 °C, shaking at 250 rpm overnight, and harvested by centrifugation at 4,000 × g, 4 °C, for 15 min. The cell pellet was resuspended in lysis buffer (50 mM potassium phosphate, pH 7.0, 300 mM NaCl, and 0.1 mM PLP). Cell disruption was performed by sonication for a 1 min burst with 4 min cooling intervals on ice for 4 cycles. Protein purification was performed with a Next-Generation Chromatography (NGC) system from BioRad (Figure S1). Cell debris was removed by centrifugation at 4 °C, 4,000 × g for 60 min, and the clarified supernatant was applied to a column containing Ni^2+^-affinity resin. The resin was then washed with 50 mM potassium phosphate, pH 7.0, 300 mM NaCl, and 0.1 mM PLP until the absorbance at 280 nm returned to baseline. The ODC was eluted with 50 mM potassium phosphate, pH 7.0, 300 mM NaCl, and 0.1 mM PLP with a linear imidazole gradient from 20–200 mM. The peak protein fractions were collected and concentrated using a centrifugal concentrator from Pall (30 kDa cutoff). To remove imidazole, the concentrated protein was repeatedly diluted with imidazole-free lysis buffer and concentrated. The final purified protein was aliquoted and stored at −80 °C. The enzyme concentration was determined by a Nanodrop (Thermo Fisher Scientific) using the absorption coefficient of 0.96 cm^–1^ for 1 mg/mL, calculated from the amino acid sequence.

### Other Enzymes

d-Ornithine decarboxylase (DOKDC) was prepared as previously described.^17^ Preparation of the C387A variant of DOKDC will be described elsewhere (S. Blankenship and R. S. Phillips, unpublished). Histidine decarboxylase was cloned from *Enterobacter aerogenes* in pLATE11, expressed in *E. coli* BL21­(DE3), and purified as described by Kamath et al.^22^

### DNBS Assay of ODC and HDC

The assay was performed as follows:

1 Ornithine decarboxylase (ODC) or histidine decarboxylase (HDC), at a concentration of 5.0 μg/mL, was added to 50 mM Bis–Tris buffer (pH 6.5) containing 1.2 mM l-ornithine or l-histidine and 16 μM pyridoxal-5’-phosphate (“PLP”), in a final volume of 250 μL in a 1.5 mL microcentrifuge tube.
2 Following a 5-min incubation at 37 °C, 250 μL of 0.1 M NaOH (33 mM final concentration) and 250 μL of 40 mM DNBS (13 mM final concentration) were added to the microcentrifuge tube, and the mixture was heated at 70 °C for 1 h in a dry sand bath incubator. One hour at 70 °C was found to be the optimum time for the reaction of DNBS with putrescine.
3 Toluene (750 μL) was then added to the reaction mixture in the microcentrifuge tube. It was vortexed, centrifuged for 1 min, and the upper toluene layer was removed with a transfer pipette. The absorbance scan of the toluene layer from 700 to 300 nm was then measured in a semi-micro quartz cuvette in a Cary 100 UV-visible spectrophotometer. The absorbance at 340 nm was used to determine the concentration of the decarboxylation product.

Standards were run under the same conditions, with the addition of 0.16 mM putrescine. To investigate the kinetic properties of ODC, the concentration of l-ornithine was varied, and the data were analyzed in GraphPad Prism using the Michaelis–Menten equation (eq 1) to fit hyperbolic curves.

1 $$V=\frac{V_{max}\times \left[S\right]}{K_{m}+\left[S\right]}$$

### Data Analysis

Each experiment was performed in triplicate. GraphPad Prism was used to generate calibration curves and perform kinetic analyses for ODC, while Origin was used for spectral analysis. The limit of detection (LOD) and limit of quantification (LOQ) were calculated using the equations LOD = 3.3 σ/m and LOQ = 10 σ/m, where σ represents the standard deviation of the blank, and *m* is the slope of the calibration curve.

## Results and Discussion

To validate the reliability of the DNBS assay, putrescine was used as a standard to quantify ODC activity. After incubation with DNBS, the toluene-extracted putrescine derivative produces a distinct absorbance peak at 340 nm due to the resulting 1,6-bis­(2,4-dinitrophenyl) putrescine (Figure 2A and Figure S6). In the presence of ODC and l-ornithine, the peak at 340 nm is observed, which is linearly dependent on the concentration of ODC, whereas no absorbance appears at this wavelength in the absence of ODC, indicating that no putrescine is formed (Figure 2A). LC–MS analysis confirmed the formation of the expected DNBS-derivatized product (C_16_H_16_N_6_O_8_), exhibiting a protonated molecular ion [M + H]^+^ at m/z 421.1087 (predicted m/z 421.1109; Figure S6) in samples containing ODC and substrate, and putrescine as a positive control. Toluene was selected as the extraction solvent since it has been used previously for assays with TNBS.^13^ Older procedures used benzene as the extraction solvent,^23^ but toluene has replaced benzene recently due to reduced toxicity. We found that toluene efficiently extracted the DNBS product, yielding a distinct absorbance peak at 340 nm. In addition, the toluene–buffer system achieved approximately 75% extraction efficiency compared with extraction using toluene alone (Figure S3). As shown in Figure 2B, the DNBS assay provided comparable sensitivity to conventional 5% water-soluble TNBS. This demonstrates that DNBS is a practical alternative to TNBS for the ODC activity assay, offering similar convenience and much lower cost (∼$0.01 per assay) while maintaining comparable accuracy and precision. In addition, background interference studies were conducted for Bis–Tris (tertiary amine), Bis–Tris propane (secondary amine), Tris (primary amine), HEPES, and sodium pyrophosphate, as presented in the supporting Information (Figure S2). The results showed that these buffers, across different pH conditions, did not generate significant interference with the ODC assay. Based on optimization experiments, ODC exhibited the highest activity in Bis–Tris buffer at pH 6.5; therefore, Bis–Tris was selected for subsequent kinetic studies. We also investigated the substrate carryover effect and its influence on the signal. Our results showed that a high substrate concentration (8 mM) with the DNBS assay does not interfere with the signal at 340 nm (Figure S4). ODC plays an essential role in both pH regulation and polyamine biosynthesis, making sensitive detection methods highly valuable. From Figure 2A, the DNBS assay shows high sensitivity for detecting ODC activity in solution, with a linear range from 0.26 to 5.2 μg/mL (Y=0.19*X+0.06, R^2^ = 1) and a low limit of detection (LOD) of 0.21 μg/mL (5.1 pmol), with an LQD of 0.64 μg/mL (15.5 pmol). The DNBS assay demonstrated good performance in detecting ODC in cell lysate samples. Even when the ODC cell lysate was diluted 100-fold, a clear and measurable signal was still observed (Figure S5). These results confirm that the DNBS assay provides reliable, sensitive monitoring of ODC activity.

**Figure 2:**
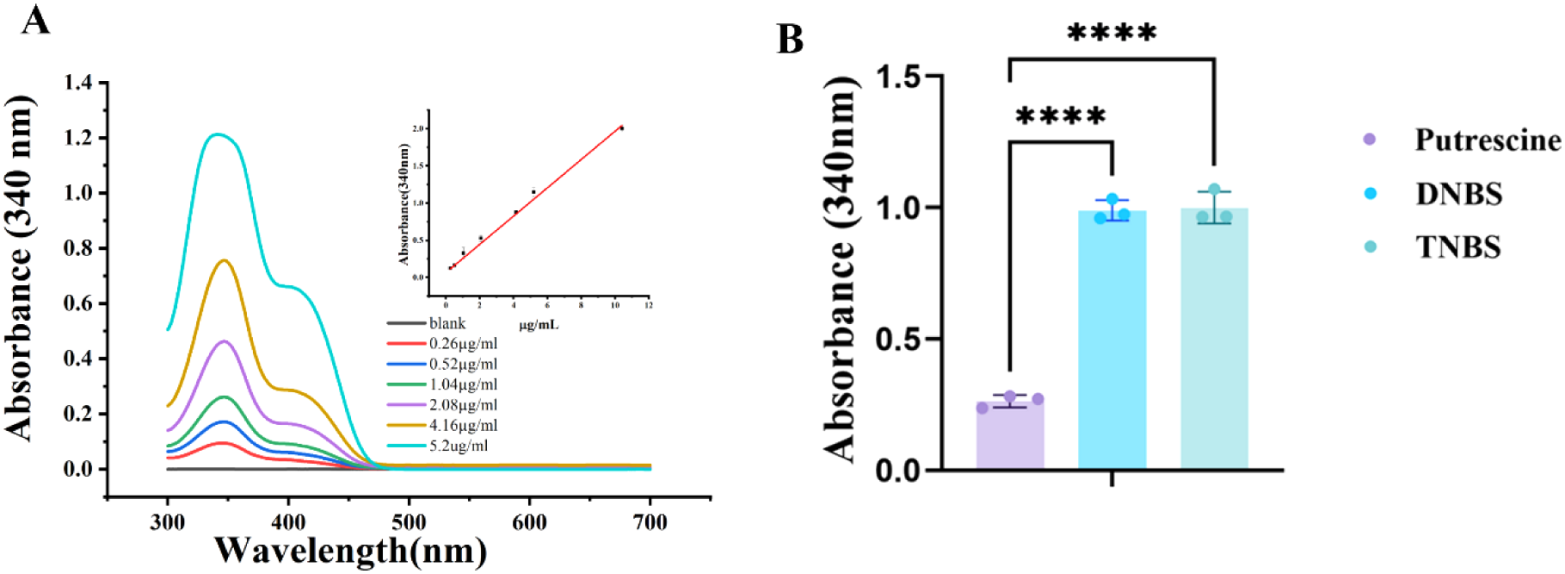
A. The UV-visible absorption spectra of ODC reactions with concentrations from 0 to 5.2 μg/mL assayed with DNBS; the inset shows the linear relationship between ODC concentrations and absorption intensity. B. The comparison of DNBS and TNBS ODC assays with putrescine as control.

Previous studies have shown that the inducible ODC contributes to pH stress regulation in bacteria. To investigate its response mechanism, we examined the catalytic kinetics of inducible ODC from *S. typhimurium* using the DNBS assay. Prior to kinetic analysis, we optimized the assay conditions by varying the incubation time of ODC with l-ornithine and optimized enzyme concentrations (Figure 3A, Figure S7). Initial-rate conditions were validated by time-course experiments showing linear product formation (Y=0.2*X+0.06, *R* ^2^ =0.99) over the first 7 min. A 5-min incubation time and an enzyme concentration of 5.0 μg/mL were selected for subsequent kinetic studies. Kinetic analysis showed that *S. typhimurium*-inducible ODC follows Michaelis–Menten kinetics (Figure 3B), with a *K*_*m*_ of 0.34 ± 0.01 mM. Furthermore, the *k*_*cat*_ of *S. typhimurium* inducible ODC was determined to be 32 ± 0.33 s^–1^, reflecting rapid substrate turnover. This high catalytic efficiency (*k*_cat_/*K*_m_ = (9.4 ± 0.35) × 10^4^ M^–1^ s^–1^) supports its role in consuming protons (H^+^) to counteract pH stress on the cell.

**Figure 3:**
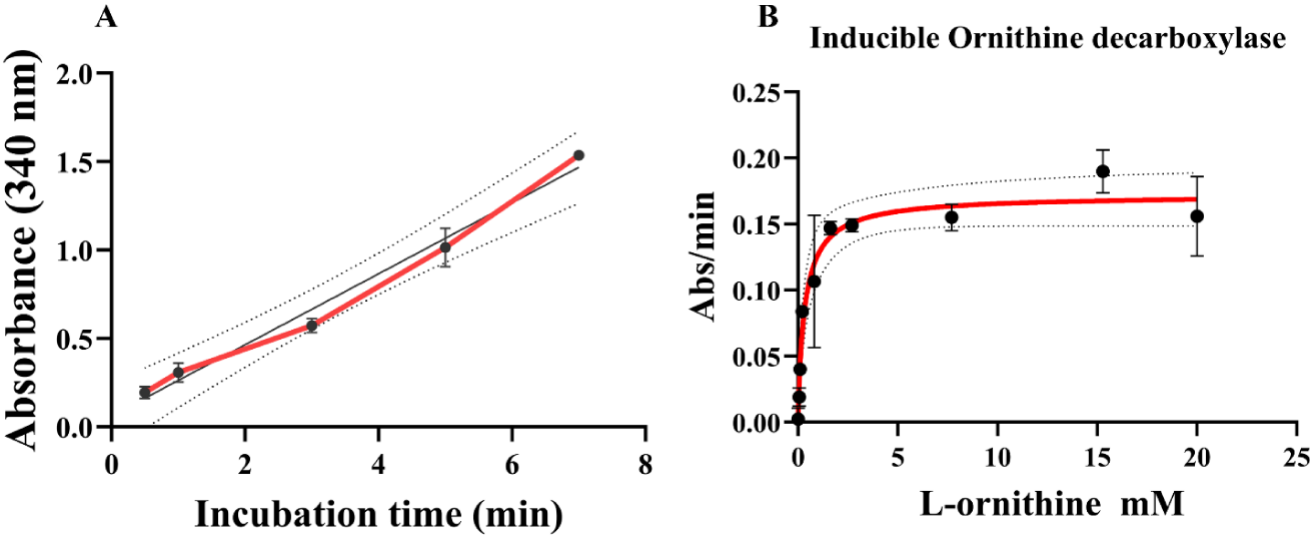
A. ODC reaction time optimization. B. Effect of ornithine concentration on the rate of ODC. The line represents the fit to the Michaelis–Menten equation (eq 1), with the parameters given in the text.

Polyamines also play a critical role in cellular biosynthesis, with putrescine representing the first committed step in polyamine production. Moreover, putrescine and cadaverine are also produced by microbes during food spoilage, making them important indicators of food quality and safety. Histamine is another biogenic amine of concern in the food industry, as its accumulation can cause abdominal cramps, hypotension, and allergy-like reactions. Moreover, putrescine and cadaverine act synergistically with histamine, enhancing its toxicity and contributing to food poisoning and other health risks.^24^ In this study, we demonstrated that the DNBS assay provides a cost-effective and straightforward alternative for detecting not only putrescine but also histamine. As shown in Figure 4B, the assay exhibited a robust linear response for putrescine over a range of 0–160 nmol (Y = 0.005*X + 0.02, R^2^=0.992), with an LOD down to 12 nmol and an LOQ of 36 nmol. Similarly, Figure 4C shows the spectrum of assays where histidine decarboxylase (HDC) was incubated with l-histidine to produce histamine. In the absence of HDC, no absorbance was detected, showing that no histamine was produced. Figure 4D shows that the DNBS assay achieved decent histamine detection, with a linear range of 0–160 nmol (Y = 0.0055*X + 0.0059, R^2^ = 0.992) and an LOD of 18 nmol and an LOQ of 56 nmol.

**Figure 4:**
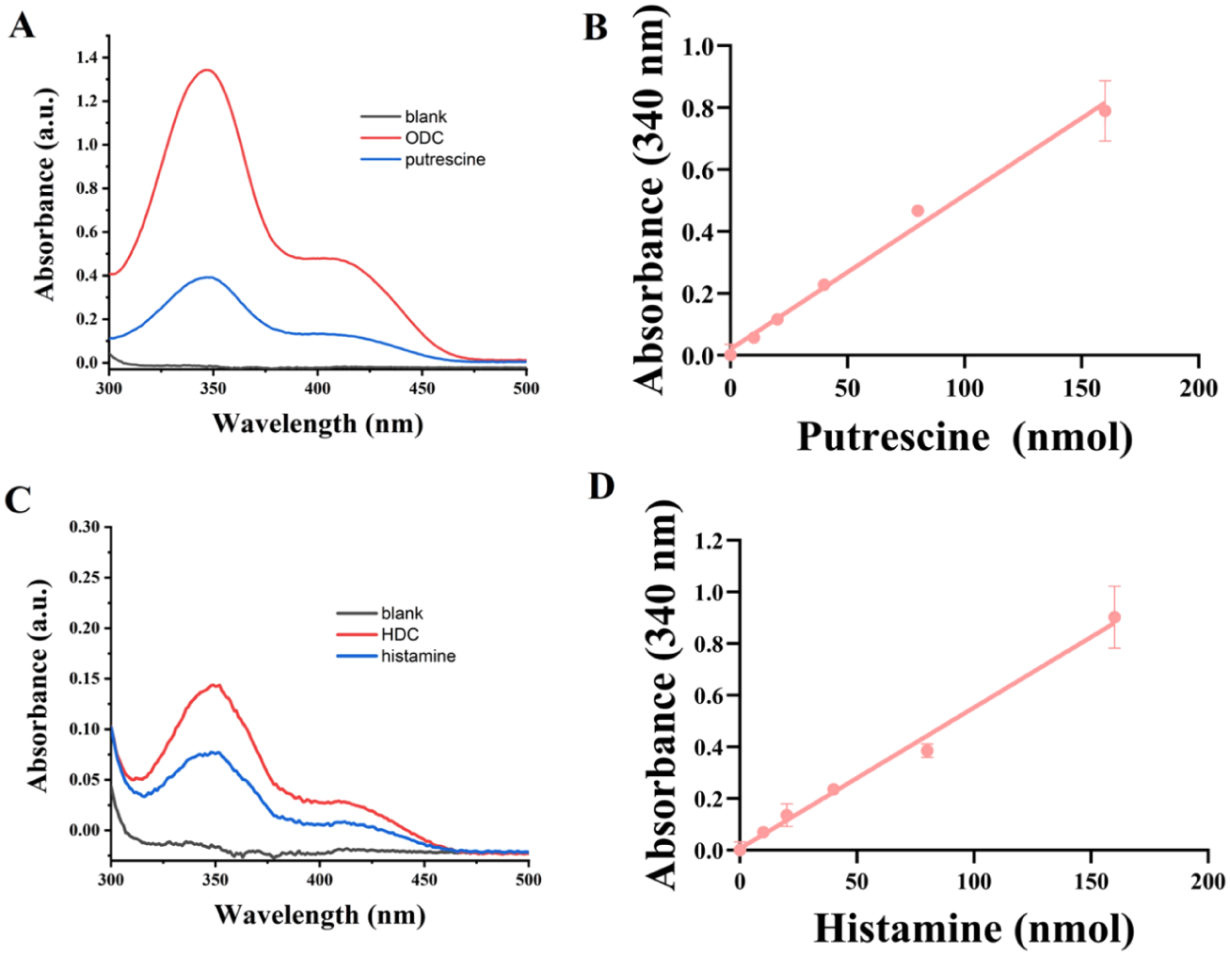
A. UV-visible absorption spectra of ODC activity with putrescine as the control. B. Linear relationship of the DNBS assay to detect putrescine. C. UV-visible absorption spectra of HDC activity with histamine as the control. D. Linear relationship of using DNBS to detect histamine.

Decarboxylases are essential for both eukaryotic and prokaryotic cellular processes. Developing a universal assay to quantify their activities has been challenging. Although a rapid method using modified ninhydrin after chloroform extraction was introduced in 1971 to detect NPY, it was not until 1980 that the first commercial ELISA kit for NPY detection was developed.

Ornithine and lysine decarboxylases still necessitated 1–4 h of incubation or overnight cultures, rendering it laborious.^25^ The DNBS-based assay overcomes these limitations, offering a rapid, cost-effective alternative with only 1 h of incubation. Application of the DNBS assay (Figure 5) demonstrated its effectiveness for DOKDC and the low-activity mutant C387A DOKDC. DOKDC incubated with d-ornithine produced significant amounts of putrescine, detectable at 340 nm following toluene extraction. The C387A DOKDC mutant has much lower activity (∼1%), but the DNBS assay allowed accurate measurement of the kinetics with both d-ornithine and d-lysine, showing the sensitivity of the assay. Furthermore, the DNBS assay of incubations of DOKDC and C387S DOKDC with d-arginine demonstrated that d-arginine is a substrate, which was not observed previously since its activity is only about 1% that of d-ornithine. Thus, the DNBS assay described here can be used to measure the activity of ornithine, lysine, arginine, and histidine decarboxylases.

**Figure 5:**
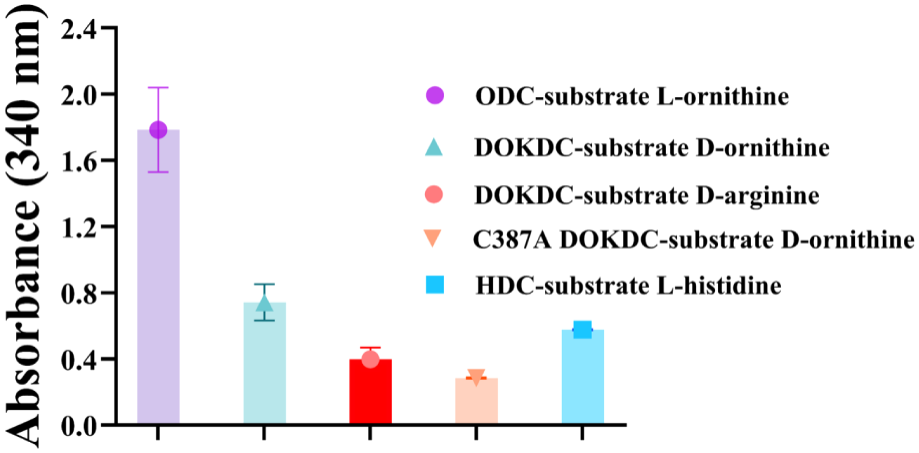
DNBS assay for the detection of decarboxylase enzyme activity. For C387A DOKDC, the enzyme concentration was 100 μg/mL, and the sample was incubated for 1 h at 37 °C.

## Conclusions

We have shown that the DNBS assay provides an economical and convenient method for detecting ODC activity, which demonstrated a linear detection range of 0.26–5.2 μg/mL for inducible ODC from *Salmonella* and exhibiting sensitivity for measuring cell lysate samples with a 100-fold. The DNBS assay also proved effective for histamine, with a good linear range and an LOD down to 18 nmol, indicating its suitability for potential applications in the food industry. Furthermore, the assay was applied to DOKDC, where the mutant C387A DOKDC, with much lower activity, could be easily assayed. Collectively, these results highlight the DNBS assay as a versatile, precise, and rapid platform for monitoring decarboxylase activity across diverse enzymes and substrates, with potential applications in biological research.

## Supplementary Material



## Notes

Purification of ODC; interference from buffers; extraction efficiency; substrate interference; assay in crude cell lysates; identification of the DNBS reaction product; effect of incubation time; and product recovery (PDF)
