# Supplementary material for: A Convenient and Economical Spectrophotometric Assay for Ornithine Decarboxylase and Related Amino Acid Decarboxylases Using Sodium 2,4-Dinitrobenzenesulfonate
Source: ACS Omega. 2026 Jun 10;11(24):35124–9. doi: 10.1021/acsomega.5c12564 (PMC13294871; doi:10.1021/acsomega.5c12564)
Supplement: Supplementary file 1 [file ao5c12564_si_001.pdf]

A Convenient and Economical Spectrophotometric Assay for Ornithine Decarboxylase and Related Amino Acid Decarboxylases using Sodium 2,4-Dinitrobenzenesulfonate

Caihong Li[a], Sara Blankenship[a], Nikki Zheng[b] , Robert S. Phillips [a,b]*

[a] Department of Chemistry, University of Georgia, Athens, GA, 30602, USA

[b] Department of Biochemistry and Molecular Biology, University of Georgia, Athens, GA, 30602, USA

*Corresponding author. Department of Chemistry, University of Georgia, Athens, GA, 30602, USA. Email: plp@uga.edu

Content

Figure S1. Purification chromatogram of ODC.

Figure S2. Assessment of DNBS assay background interference from various buffer compositions.

Figure S3. Comparison of putrescine extraction using toluene alone versus buffer–toluene extraction.

Figure S4. Evaluation of substrate interference with DNBS assay background signal.

Figure S5. Application of the DNBS assay to analyze ODC cell lysate samples.

Figure S6. LC–MS validation of the DNBS derivative.

Figure S7. Extended incubation time of ODC.

Table S1. Recovery experiment of spiked putrescine and histamine.

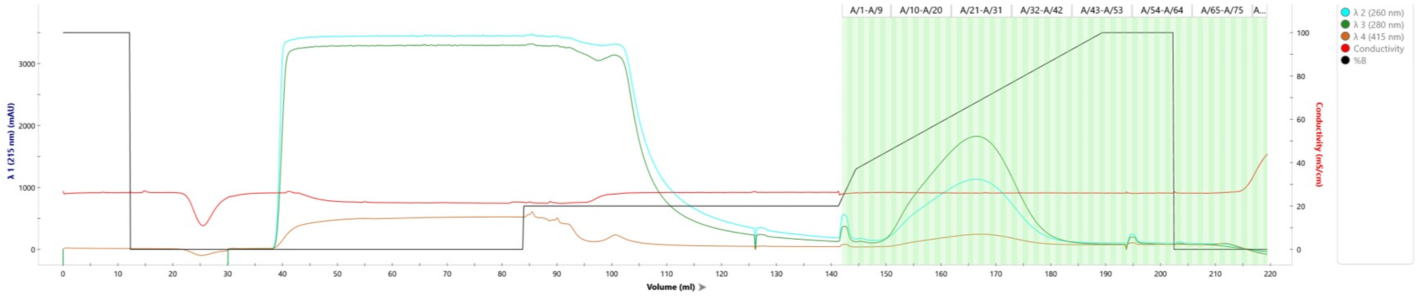

Figure S1. Purification chromatogram of ODC illustrating column equilibration and washing, loading of the crude sample, elution with an imidazole-containing resin, and subsequent fraction collection (see Materials and Methods for purification details).

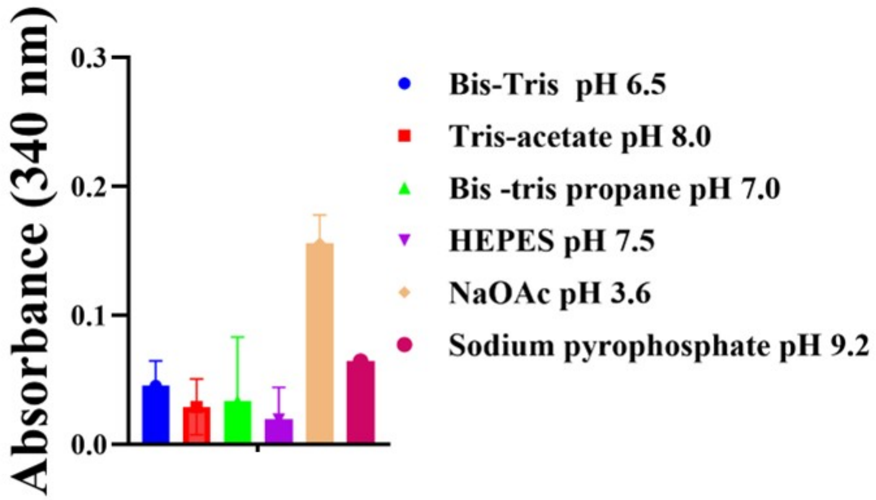

Figure S2. Evaluation of background signals caused by different buffers using DNBS assay. Buffers were prepared at a concentration of 50 mM and incubated with 33 mM NaOH and 13 mM DNBS in a microcentrifuge tube, followed by heating at 70 °C for 1 h in a dry bath incubator to terminate the reaction. The reaction products were extracted with toluene, and absorbance was measured at 340 nm.

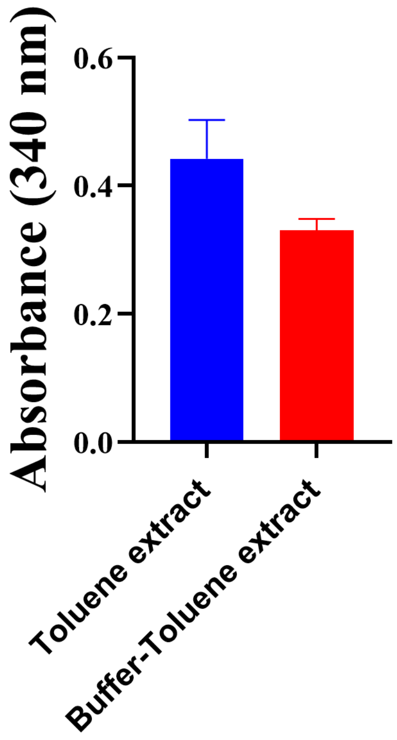

Figure S3. Comparison of putrescine extraction using toluene alone versus buffer–toluene extraction. For toluene-only extraction, 0.16 mM putrescine was incubated with 33 mM NaOH, 13 mM DNBS, and 750 µL toluene, followed by heating at 70 °C for 1 h in a dry bath incubator. For buffer–toluene extraction, buffers (50 mM Bis-Tris, pH 6.5) containing 0.16 mM putrescine were incubated with 33 mM NaOH and 13 mM DNBS in microcentrifuge tubes and heated at 70 °C for 1 h to terminate the reaction, after which 750 µL toluene was used for extraction. Absorbance was measured at 340 nm.

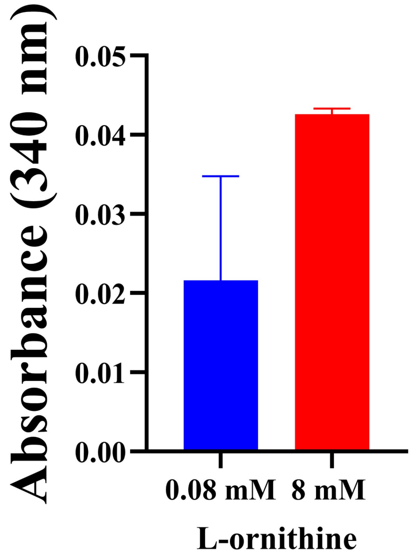

Figure S4. Substrate interference with background signals was evaluated. Buffers (50 mM, Bis-Tris pH 6.5) containing the substrate L-ornithine at concentrations of 0.08 and 8 mM were incubated with 33 mM NaOH and 13 mM DNBS in microcentrifuge tubes, followed by heating at 70 °C for 1 h in a dry bath incubator to terminate the reaction. The reaction products were extracted with toluene, and absorbance was measured at 340 nm.

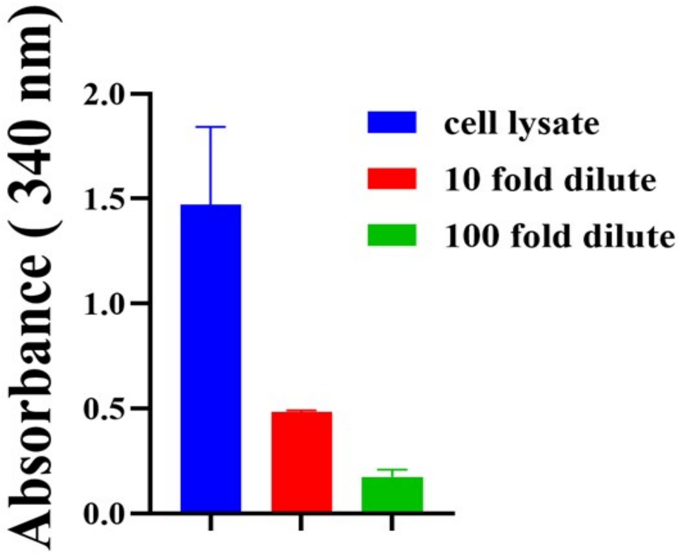

Figure S5. Application of the DNBS assay for analysis of ODC activity in cell lysate samples. The procedure was identical to that used for the standard ODC activity assay.

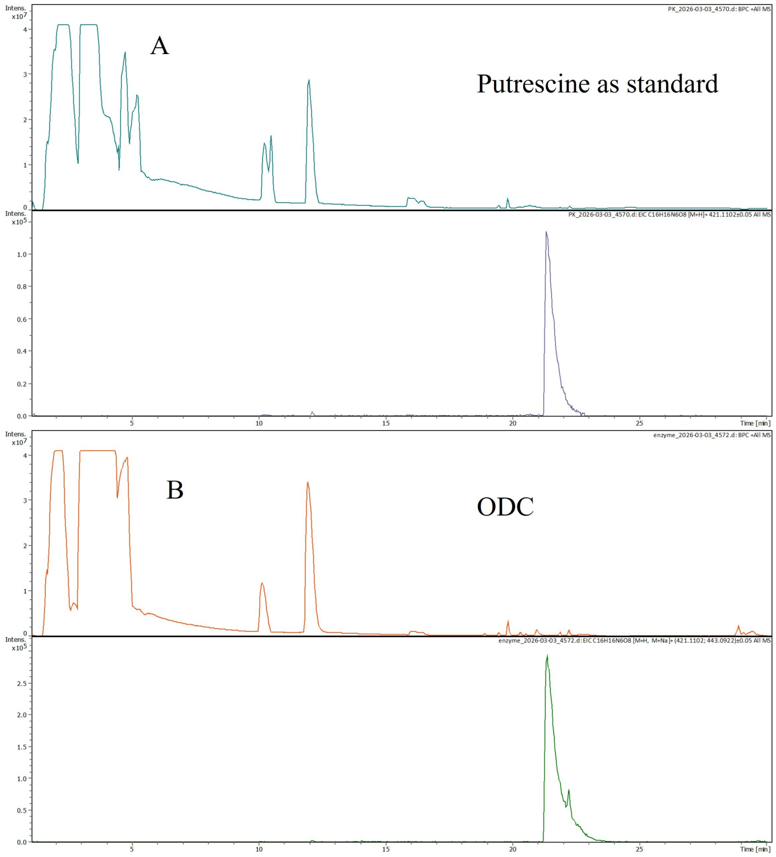

Figure S6. (A) LC-MS data for DNBS-reacted putrescine standards used as a negative control (B) ODC-treated ornithine sample derivatized with DNBS. For panel A, putrescine (320 μM) was added to 33 mM NaOH and 13 mM DNBS in microcentrifuge tubes, followed by heating at 70 °C for 1 h in a dry bath incubator to terminate reaction. For panel B, the procedure was identical to that used in the ODC activity assay followed by LC–MS analysis. The Molecular weight of 2,4-Dinitrobenzenesulfonate is $C_{16}H_{16}N_6O_8$ (420 Da).

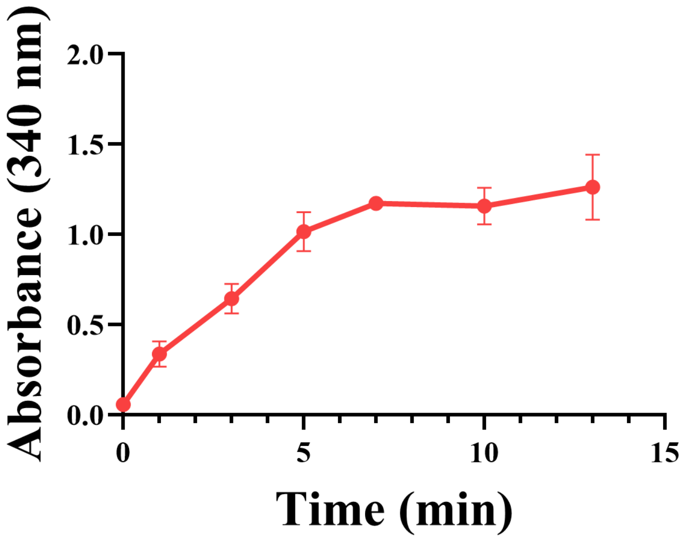

Figure S7. Time-course analysis of ODC incubation using the DNBS assay. The procedure followed the same protocol as the standard ODC activity assay.

**Sipke / nmol**	**40**	**80**	**160**
**Putrescine recovery %**	**87.8 ± 3.6**	**65.6 ± 3.0**	**62.3 ± 2.5**
**Sipke / nmol**	**20**	**40**	**80**
**Histamine recovery %**	**53.3 ± 6.2**	**66.4 ± 1.1**	**59.3 ± 0.2**

Table S1. Spike–recovery experiments were performed for putrescine by spiking known amounts (40, 80, and 160 nmol) of putrescine and (20, 40, and 80 nmol) of histamine into 50 mM Bis-Tris buffer (pH 6.5) containing 1.2 mM L-ornithine and 16 µM PL. The samples reacted with 33 mM NaOH and 13 mM DNBS to a final volume of 250 µL in microcentrifuge tube and heated at 70 °C for 1 h to terminate reaction. After 750 µL of toluene was added for extraction. Absorbance was measured at 340 nm. Percent recovery was calculated as $(C_{spiked}-C_{blank})/C_{added}\times100\%$, C_{added} is known spike concentration. All experiments were performed in triplicate using three independent biological replicates.
